# Supplementary material for: Molecular Insights into the Superiority of Platelet Lysate over FBS for hASC Expansion and Wound Healing
Source: Cells. 2025 Jul 25;14(15):1154. doi: 10.3390/cells14151154 (PMC12346565; doi:10.3390/cells14151154)
Supplement: Supplementary file 1 [file cells-14-01154-s001.zip › cells-3729380-supplementary.pdf]

Supplementary Information

Molecular insights into the superiority of platelet lysate over FBS for hASC expansion and wound healing

Sakurako Kunieda[1], Michika Fukui[1], Atsuyuki Kuro[2], Toshihito Mitsui[1], Huan Li[1], Zhongxin Sun[1], Takayuki Ueda[1], Shigeru Taketani[1], Koichiro Higasa[3], and Natsuko Kakudo[1*]

[1]Department of Plastic and Reconstructive Surgery, Kansai Medical University, 2-5-1 Shin-machi, Hirakata, Osaka 573-1010, Japan.

[2]Department of Plastic and Reconstructive Surgery, Kansai Medical University Medical Center, 10-15, Fumizono-cho, Moriguchi, Osaka 570-8507, Japan.

[3]Department of Genome Analysis, Institute of Biomedical Science, Kansai Medical University, 2-5-1 Shin-machi, Hirakata, Osaka 573-1010, Japan.

Correspondence author: Natsuko Kakudo
Department of Plastic and Reconstructive Surgery, Kansai Medical University, 2-5-1 Hirakata, Osaka 573-1010, Japan, Tel.: +81-72-804-0101, Fax: + 81-72-804-2031
E-mail: kakudon@hirakata.kmu.ac.jp

Table S1. Primer sequences used for qPCR analysis.

Gene	Forward Primer (5′ → 3′)	Reverse Primer (5′ → 3′)
**GAPDH**	GTCTCCTCTGACTTCAACAGCG	ACCACCCTGTTGCTGTAGCCAA
**FANCA**	CAGAACCCAACTCTGCTGAGGA	ATCACTGCCACCTGTGCCGATA
**FEN1**	ACTAAGCGGCTGGTGAAGGTCA	GCAGCATAGACTTTGCCAGCCT
**MT2A**	GAGTGCAAATGCACTTCGTGCAA	GCGTTCTTTACATCTGGGAGCG
**MCM5**	GACTTACTCGCCGAGGAGACAT	TGCTGCCTTTCCCAGACGTGTA
**CDC20**	CGGAAGACCTGCCGTTACATTC	CAGAGCTTGCACTCCACAGGTA
**RAD18**	GGATTGTCCTGTTTGCGGGGTT	GTTTTGGGCAGCGGCTTCCTTT
**CHEK1**	GTGTCAGAGTCTCCCAGTGGAT	GTTCTGGCTGAGAACTGGAGTAC
**IL6**	AGACAGCCACTCACCTCTTCAG	TTCTGCCAGTGCCTCTTTGCTG
**IL1B**	CCACAGACCTTCCAGGAGAATG	GTGCAGTTCAGTGATCGTACAGG

Figure S1

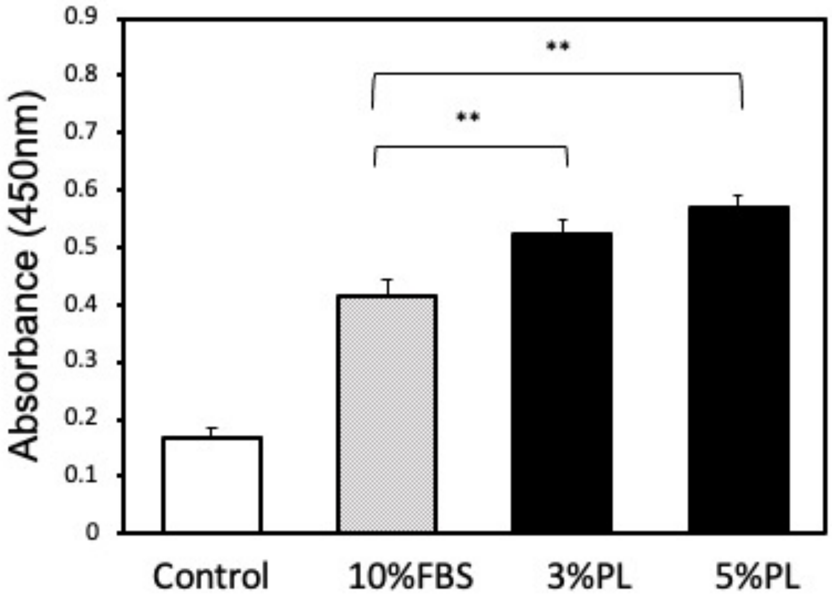

Figure S1 | Effect of PL on hASCs proliferation. Cells were cultured in DMEM without (control) or with 10% FBS, 3% PL and 5% PL. At the 48h-cultivation, cells were incubated with CCK8 reagent. Absorbance at 450nm was measured (**P<0.01 between the indicated group). Values are presented as means ± SD of 5 experiments.

Figure S2

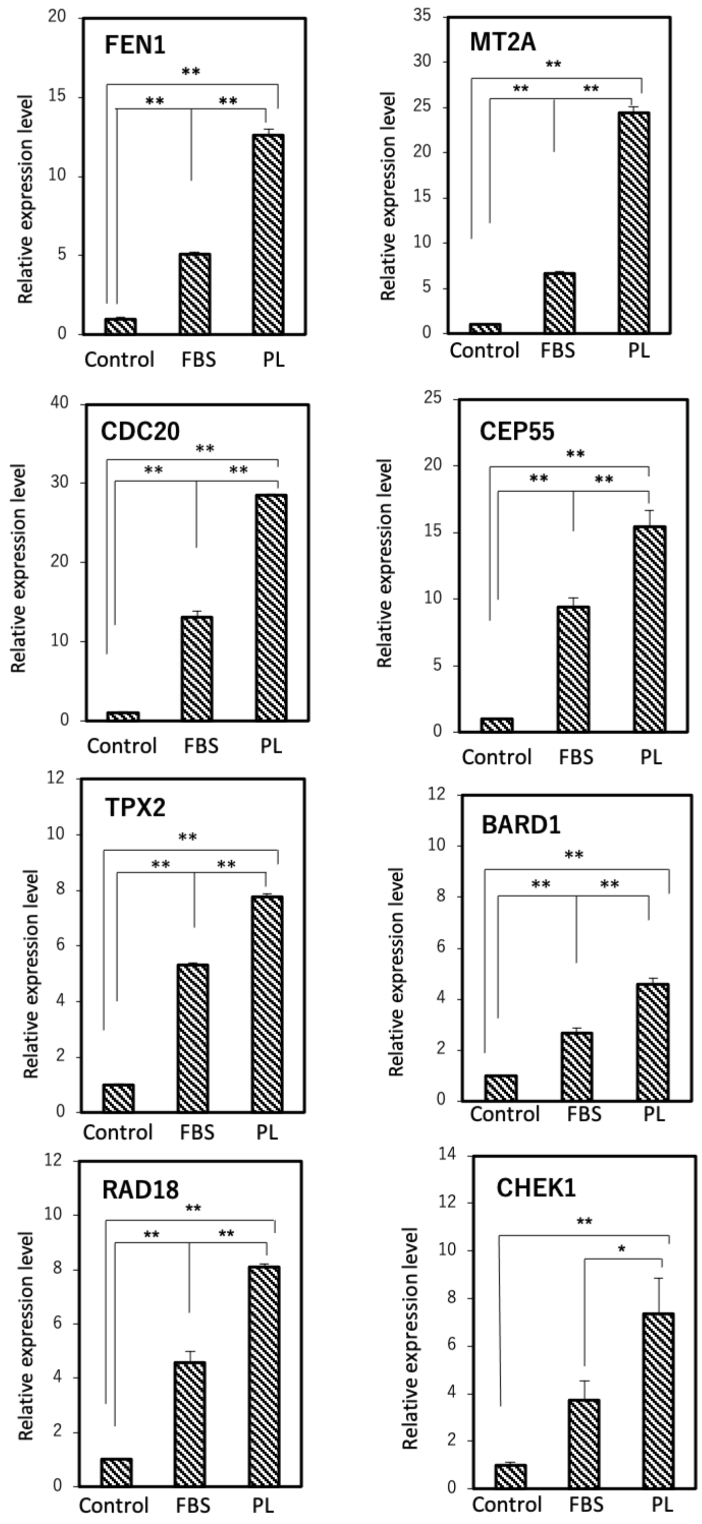

Figure S2 | Representative gene expression in hASCs cultured without or with 10% FBS or 3% PL. The relative expression levels of cell proliferation (FEN1, MT2A, CDC20, CEP55 and TPX2) and stress resistance (BARD1, RAD18 and CHEK1)-related genes.

Figure S3

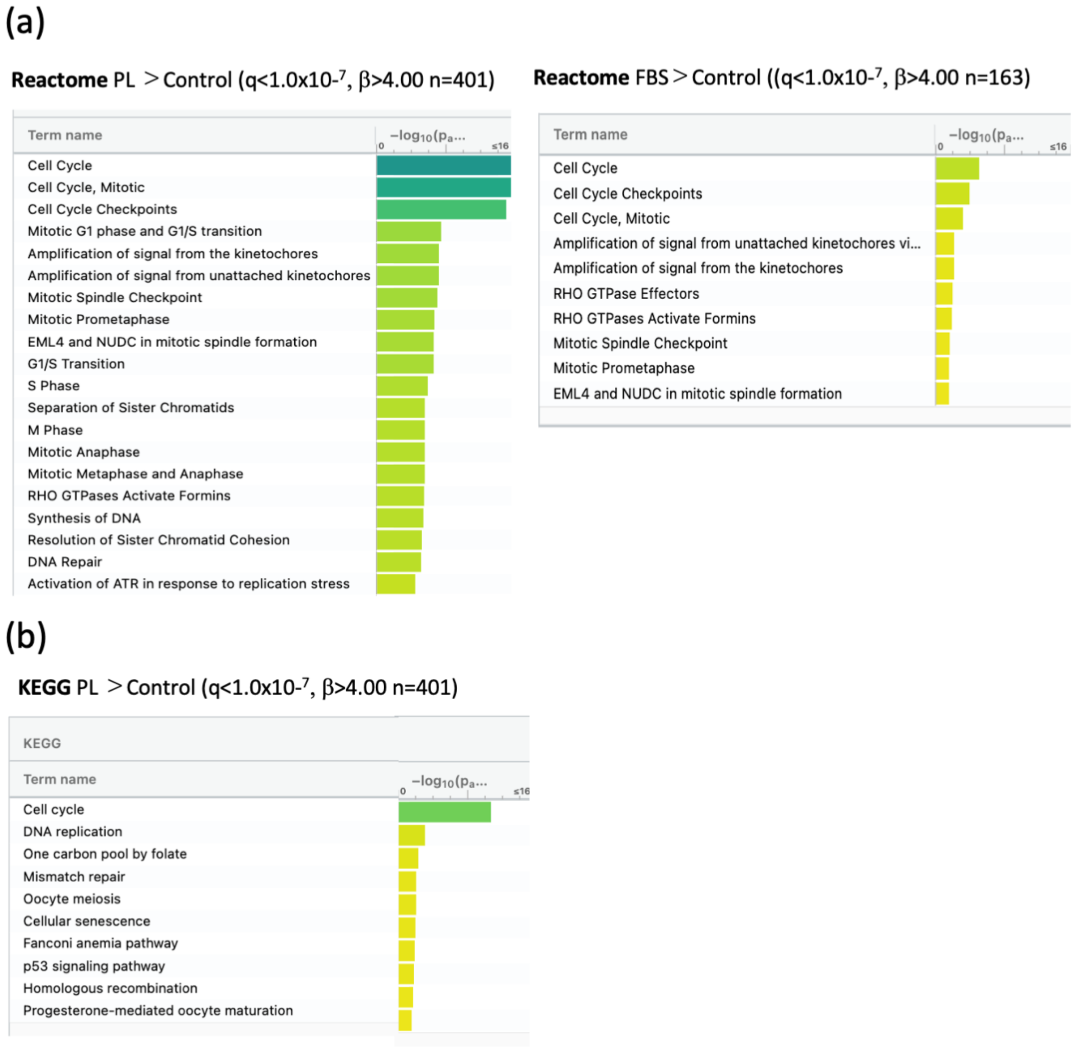

Figure S3 | Pathway over-representation analysis (ORA) of differentially expressed genes ($q < 10e^{-7}$, $\beta > 4.00$). (a) Reactome pathways for PL vs Control and FBS vs Control.
(b) KEGG pathways for PL vs Control. No KEGG pathways met the threshold in the FBS vs Control comparison.

Figure S4

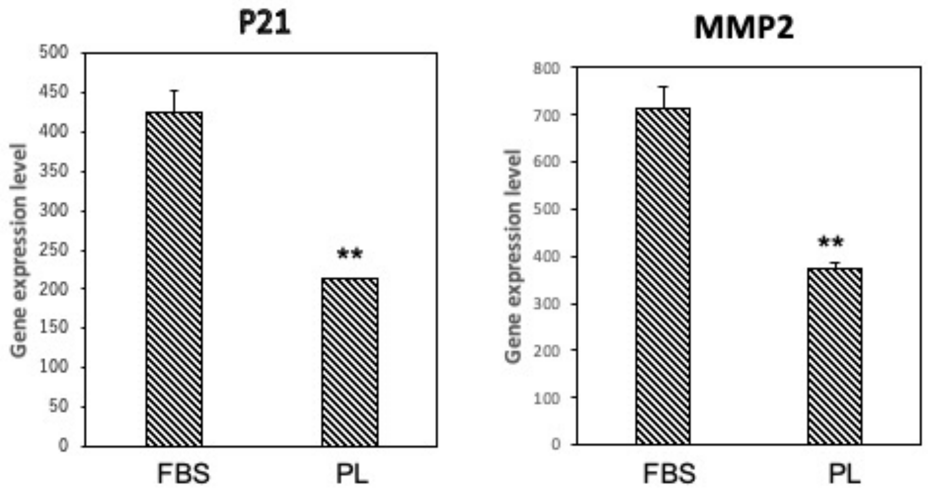

Figure S4 | Expression of p21 and MMP2 mRNAs in hASCs cultured with 10% FBS or 3% PL. The gene expression levels of cellular senescence (p21 and MMP2). (**$P<0.01$ between two groups). Values are expressed as means ± SD (n=3)

Figure S5

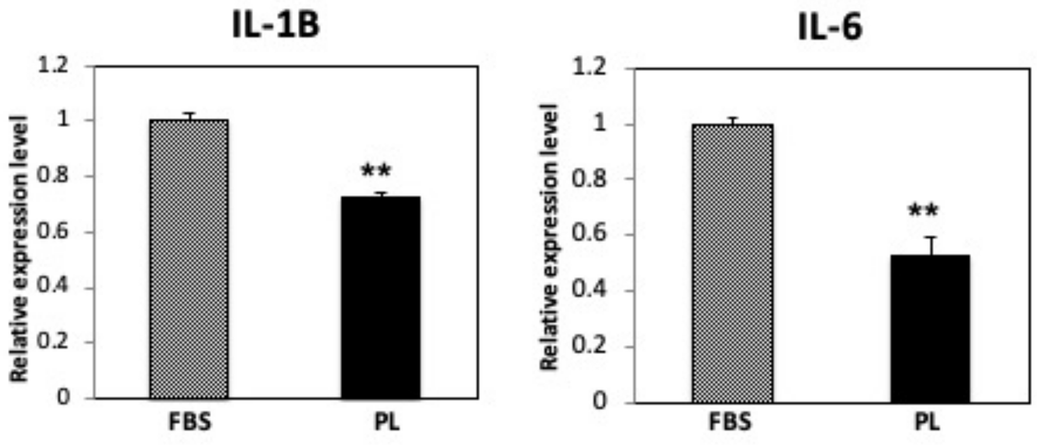

Figure S5 | RT-qPCR analysis of IL-1β and IL-6 in hASCs cultured with FBS or PL. RNA was isolated from hASCs cultured with DMEM+10% FBS or 3% PL for 48h. RT-qPCR was performed. Values are expressed as means ± SD (n=3), **P<0.01 between two groups.
